# The impact of environmental variations (temperature and humidity) on *Fusarium graminearum* in broiler and pig feed

**DOI:** 10.3389/ffunb.2026.1810614

**Published:** 2026-04-24

**Authors:** Osias Thabo Ramoeletsi, Olerato Tumiso Sebolao, Martha Cebile Jobe, Mulunda Mwanza

**Affiliations:** 1 Department of Animal Health, North-West University, Mmabatho, South Africa; 2 Food Security and Safety Niche, Faculty of Natural and Agricultural Sciences, North-West University, Mmabatho, South Africa

**Keywords:** broiler and pig feed, *Fusarium*, moisture content, mycotoxin, temperature

## Abstract

Fusarium-related mycotoxins are a major concern in livestock feeds due to their toxicity and potential for co-occurrence, which can cause additive or synergistic effects. This study investigated the influence of temperature and moisture content on fusarium-associated mycotoxin production in pig and broiler feeds. Feeds were inoculated with the *Fusarium graminearum* and incubated under controlled temperatures (30-40 °C) and moisture levels (8-15%), and mycotoxins including deoxynivalenol (DON), Fusarenone-X (FUX), fumonisins B_1_ and B_2_ (FB_1_, FB_2_), ochratoxin A (OTA), T-2 toxin, HT-2 toxin, and zearalenone (ZEN) were quantified using LCMS. Results showed that temperature was the primary driver of mycotoxin production, while moisture content modulated mycotoxin magnitude and diversity in a feed-dependent manner. In pig feed, fumonisins were predominantly produced at 30 °C under low moisture, with FB_1_ reaching 1850 µg/kg. DON and FUX were detected across a wider temperature range, whereas ZEN remained relatively stable. OTA, T-2, and HT-2 toxins were infrequently detected. Broiler feed showed similar patterns, with DON and FUX consistently present, fumonisins largely restricted to lower temperatures, and ZEN stable across conditions. Co-occurrence of multiple mycotoxins was most pronounced at lower temperatures, highlighting the risk of chronic multi-toxin exposure. Overall, these findings emphasize the importance of optimized feed storage and multi-mycotoxin monitoring. Feed-specific mitigation strategies, including antifungal additives or plant-derived bioactive compounds, may help reduce mycotoxin accumulation and safeguard livestock health and productivity.

## 1 Introduction

Mycotoxin levels in animal feed have been reported to be higher than those in humans who eat comparatively well-refined and processed meals (Yang et al., 2020). Food, feed, and agricultural products contaminated with mycotoxin have become a serious concern because these toxic substances can cause various problems that result in a variety of health problems, from acute to chronic problems in both animals and humans (Khan, 2024). Globally, the major threats to food safety, mycotoxin contamination, remain one of the most persistent and economically damaging challenges (Latham et al., 2023). Mycotoxins are toxic secondary metabolites produced by filamentous fungi, particularly species of the genera *Aspergillus, Fusarium*, and *Penicillium*. Among the toxigenic fungi, *Fusarium graminearum* and *Fusarium verticillioides* are of particular concern due to their ability to produce highly toxic fumonisins (FBs), trichothecenes, especially deoxynivalenol (DON) and zearalenone (ZEN) (Mesterhazy, 2024; Sherif et al., 2023). These mycotoxins are commonly found in cereal-based feed ingredients such as maize, wheat, and barley, which form the backbone of broiler and pig diets. DON is known to cause feed refusal and reduced weight gain (Hooft and Bureau, 2021), and intestinal barrier dysfunction (Chen et al., 2024), especially in pigs, while ZEN exhibits strong estrogenic effects, leading to reproductive disorders, while also posing serious food safety risks through carry-over into meat products (Jing et al., 2022).

The growth and development of *Fusarium* spp is widely affected by environmental factors such as temperature and humidity (Ezrari et al., 2021; Peter Mshelia et al., 2020). Since Africa’s agricultural production is entirely dependent on weather and climatic variables, the continent is particularly vulnerable to the effects of climate change and the production of mycotoxins. These factors play a central role in the growth of *Fusarium spp*, especially in contaminating poultry and pig feed. Efficient reduction of mycotoxins in Africa will require an understanding of the various causes of elevated concentrations of these toxins in African foods (Nji et al., 2022).

Temperature has a direct influence on the growth and development of *F. graminearum.* Research has shown that the optimal temperature for growth of *F. graminearum* is between 25–30 °C, with faster growth rates observed in this temperature range (Liu et al., 2022). As temperatures increase due to climate change and seasonal variations, the risk of *F. graminearum* in crops and subsequently in feed increases. Humidity is another important environmental factor affecting *F. graminearum* growth and mycotoxin production. *F. graminearum* thrives in conditions of high relative humidity, especially during the flowering and seeding stages of cereal crops (Manstretta and Rossi, 2016; Zhu et al., 2023). The economic impact of *F. graminearum*-associated mycotoxicosis in livestock production is therefore substantial. Increasing humidity in storage facilities can also contribute to fungal growth and mycotoxin contamination in stored feed. While fusarium mycotoxin contamination in animal feeds is well documented, few studies have systematically compared pig versus broiler feed under controlled temperature and moisture gradients. Moreover, limited research has explored the interactive effects of temperature and moisture on co-occurrence patterns of multiple fusarium-related mycotoxins. This study addresses these gaps by evaluating how temperature and moisture interactions influence the production of DON, FUX, ZEN, T-2, HT-2, and OTA in different feed matrices. The selection of broiler and pig grower feed in this study was motivated by their critical role in ensuring food security, income generation, and nutritional sustainability in rural and peri-urban communities. Broiler and pig grower feeds are particularly susceptible to contamination by mycotoxins because they are typically composed of cereals such as maize and soybean meal, which are highly vulnerable to fungal infection during storage and handling (Nji and Mwanza, 2024; Yu and Pedroso, 2023). In small-scale farming settings, limitations in storage infrastructure, environmental control, and quality monitoring (Touch et al., 2024) increase the risk of fungal proliferation and subsequent mycotoxin production. This makes these feed types ideal matrices for investigating real-world contamination scenarios. This is particularly relevant in smallholder systems, where regulatory oversight may be limited, and contaminated feed may go undetected. Therefore, this study aims to investigate the impact of temperature and humidity variations on fusarium-related mycotoxin production. The findings are particularly relevant in the context of climate change, where increased temperatures and humidity can exacerbate fungal proliferation and toxin formation, threatening animal health, feed safety, and food security. It can also inform targeted interventions, including improved storage practices, feed management strategies, and the development of natural antifungal agents, ultimately contributing to safer food systems and enhanced farmer resilience.

## 2 Materials and methods

### 2.1 Materials

Chemicals such as HPLC grade acetonitrile, water, and acetic acid were purchased from Merck KGaA, Darmstadt, Germany. Filter syringe 0.22 µm (Membrane solutions, Auburn, Washington USA), potato dextrose agar (PDA) was purchased from Merck KGaA, Darmstadt, Germany.

### 2.2 Experimental design and feed preparation

**Figure 1:**
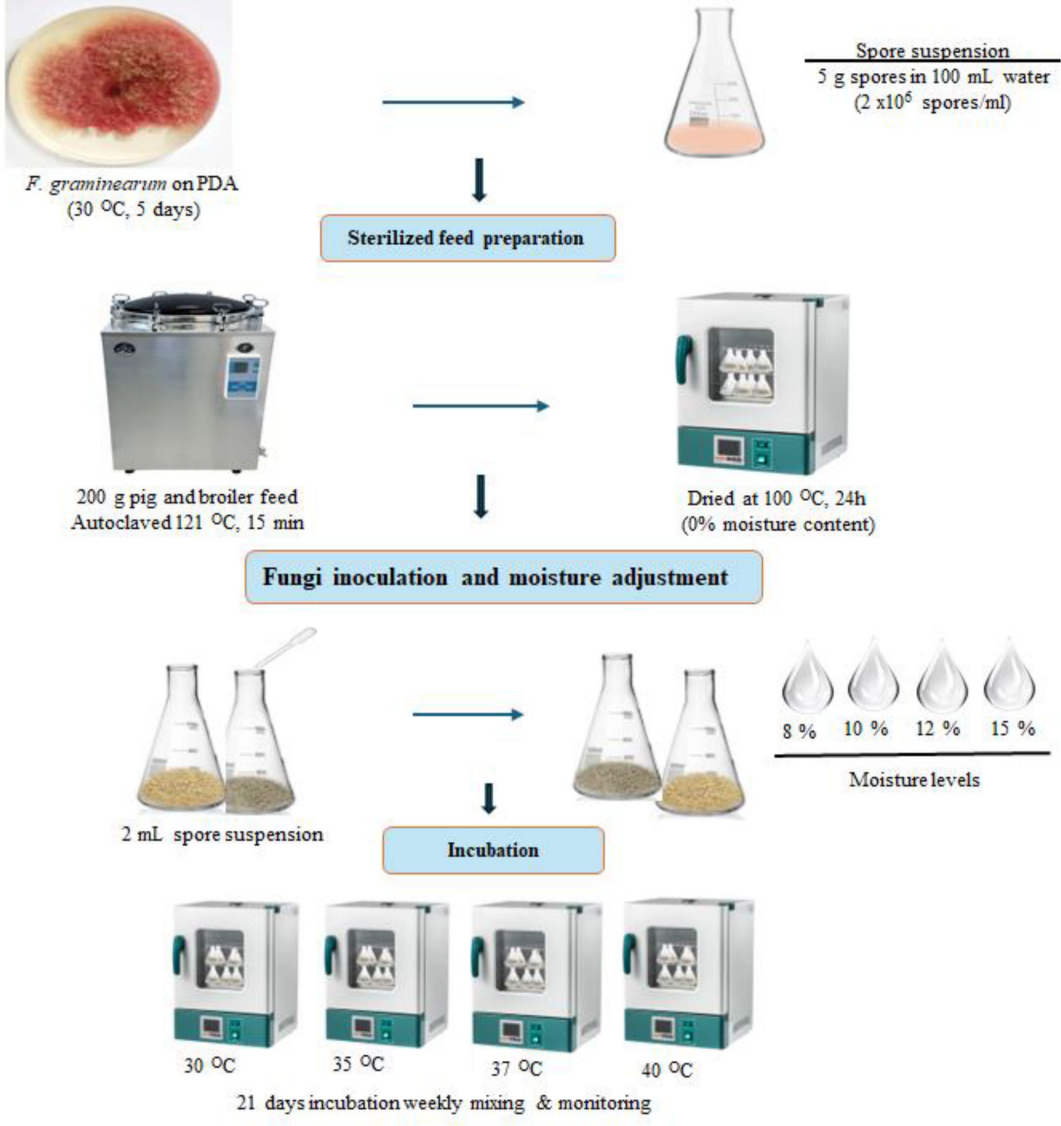
Schematic diagram showing the feed inoculation and incubation process.

The study was conducted in the Animal Health Laboratory at North-West University (Mafikeng campus). Fusarium strain was obtained from the Animal Health Laboratory and was previously identified as *F. graminearum.* The strain was revived on PDA prepared according to the manufacturer’s instructions and autoclaved at 121 °C for 15 min. The strain was inoculated at the center of the PDA plate and incubated at 30 °C for 5 days to allow sufficient mycelial growth. Spores were then harvested by weighing 5 g of fungal material and diluting it in 100 mL of sterile distilled water to prepare the inoculum. Broiler and pig grower feeds (Siyakhula PTY) used in this study were purchased at the feed store at the Mahikeng industrial site (NW, South Africa). Feed represents composite feed matrices formulated to meet the nutritional requirements of poultry and swine during early growth stages. Broiler grower typically consists of maize as the primary energy source, supplemented with soybean meal and other protein sources such as fish meal, along with vegetable oil, mineral sources, salt, and a vitamin/mineral premix. This formulation provides a crude protein content of approximately 20-23%. Pig grower feed similarly contains maize as the main energy component, combined with soybean meal, wheat bran, and small amounts of animal protein sources, minerals, and vitamin/mineral premixes, resulting in an average crude protein content of approximately 16-18% as shown in **Table 1**. The complex composition of these compounded feeds, which includes carbohydrates, proteins, lipids, and minerals, may influence fungal growth dynamics, mycotoxin production, and interactions between toxins and matrix components during extraction and analysis. Different moisture and temperature conditions were established to evaluate their influence on fungal growth and mycotoxin production. The experiment was conducted as shown in **Figure 1**, briefly, 200 g of feed samples were placed in sterilized flasks and autoclaved at 121 °C for 15 min to eliminate existing microorganisms. The feed was subsequently dried in an oven at 100 °C for 24 h to achieve 0% moisture content and to allow the samples to equilibrate prior to inoculation. Each feed sample was inoculated with 2 mL of the prepared *F. graminearum* spore suspension (2 × 10^5^ spores/mL). Sterile water was added to adjust the moisture content to 8%, 10%, 12%, or 15%. The inoculated feed samples were then incubated at controlled temperatures of 30, 35, 37, and 41 °C using a laboratory incubator, and temperature was monitored using a calibrated digital thermometer to ensure stable experimental conditions for 21 days. The selected temperature and moisture ranges were chosen to reflect environmental conditions commonly encountered during feed storage in tropical and subtropical regions, as well as to capture the threshold effects of water availability on *Fusarium* growth and mycotoxin production (Paterson and Lima, 2010). Temperatures of 30-41 °C simulate both moderate and extreme storage conditions, while moisture contents of 8-15% encompass typical feed moisture levels and conditions of improper storage that may favor fungal colonization. Samples were mixed and monitored weekly to ensure uniform growth. All experiments were conducted in four flasks, and the entire experiment was repeated three times to ensure reproducibility and statistical reliability.

**Table 1:** Feed composition for broiler and pig grower feed.

	Broiler grower	Pig grower
Ingredients %		
Corn	61.10	68.32
Soybean meal	34.60	25.00
Soybean oil	–	1.00
Dicalcium phosphate	1.50	0.80
Oyster shells	1.00	–
Meat and bone meal	–	3.35
Vitamin-mineral premix	0.50	0.10
L-lysine	0.37	0.32
NaCl	0.35	1.00
DL-methionine	0.26	0.13
Threonine	0.24	0.13
Termin 8b	0.10	–
Chemical composition (g/kg)		
Protein (min)	180	140
Methionine (min)	4.50	2.70
Moisture (max)	120	120
Fat (min)	25	25
Fibre	60	80
Calcium (min-max)	7-12	6-10
Phosphorus (min)	4.50	5

### 2.3 Extraction preparation for mycotoxin analyses

The extraction of *Fusarium* spp mycotoxins from feed samples was done following (Dada et al., 2020). Briefly, 5 g of the respective feed sample was weighed into 50 mL centrifuge tubes and extracted for 24 h at 180 rpm on a GFL 3017 rotary shaker (GFL, Burgwedel, Germany) with 20 mL of extraction solvent (acetonitrile/water/acetic acid 79:20:1, v/v/v). Then, samples were vortexed for 10 minutes and filtered through the Whatman filter paper. The filtrate was then diluted in a 1:1 ratio with a dilution solvent (acetonitrile/water/acetic acid 20:79:1, v/v/v) and transferred into amber vials through filtering with a 0.22 µm filter syringe.

### 2.4 Mycotoxin analysis using liquid chromatography mass spectrophotometry

For the analysis, Shimadzu 8050 LC-MS/MS System (Shimadzu Cooporation, Kyoto, Japan), incorporating a Turbo Ion Spray electrospray ionization (ESI) source alongside an 1100 Series HPLC System. Shimadzu C_18_ column (150 × 2.1 mm, 5 µm) (Shimadzu Cooporation, Kyoto, Japan) with mobile phase A (10 mM ammonium acetate in water) and B (2% acetic acid in methanol) kept at 50 °C was used. A binary mobile phase gradient elution at a flow rate of 0.3 mL/min^-1^ and 10 µL injection volume was used for both the standard and the samples. The ESI-MS/MS source temperature operated at 550 °C, in the multiple reaction monitoring mode, both in positive and negative polarities, in two separate chromatographic runs per sample by scanning two fragmentation reactions per analyte. Further MS parameters were as follows: curtain gas 10 psi (69 kPa of max. 99.5% nitrogen); ion source gas 1 (sheath gas) 50 psi (345 kPa of nitrogen); ion source gas 2 (drying gas) 50 psi (345 kPa of nitrogen); ion spray voltage −4000 V and +4000 V respectively, collision-activated dissociation gas (nitrogen) high. The concentration of mycotoxins was expressed as µg/kg. **Table 2** shows the MRM of different mycotoxins.

**Table 2:** Multiple reaction monitoring transitions, mass spectrometer conditions, and retention times of the determined mycotoxins.

Mycotoxin	Retention time (min)	Precursor (m/z)
Deoxynivalenol	2,410	355,10>295,10
15-acetyldeoxynivalenol	7,959	339,10>261,10
Nivalenol	1,641	371,10>281,10
Fusarenone-X	5,606	413,10>353,10
Fumonisin B_1_	9,678	722,40>334,10
Fumonisin B_2_	10,405	706,40>336,10
Fumonisin B_3_	10,404	706,40>336,10
Ochratoxin A	10,329	404,10>239,10
T-2 toxin	10,227	484,30>185,10
HT-2	9,821	442,20>263,10
Zearalenone	10,547	317,10>131,10
alpha-ZEL	10,560	319,20>275,10
Diacetoxyscirpenol	9,360	384,20>307,10

### 2.5 Statistical analysis

Data were expressed as mean ± standard deviation (SD). A two-way ANOVA was performed to evaluate the effects of temperature, moisture content, and their interaction on fungal growth and mycotoxin production.

## 3 Results

### 3.1 Mycotoxin production under different environmental conditions

#### 3.1.1 Effect of temperature and moisture on mycotoxin production

Temperature and moisture content significantly influenced mycotoxin production, with clear evidence of interaction effects between these factors for several fusarium-related toxins (**Tables 3**, **4**). The fusarium-associated mycotoxins detected were DON, FUX, FB_1_, FB_2_, OTA, T-2 toxin, and HT-2 toxin. Overall, mycotoxin production varied markedly in response to changes in temperature and moisture content. **Table 1** summarizes the effects of temperature and moisture content on fusarium-related mycotoxin production in pig feed. Fumonisins (FB_1_ and FB_2_) were predominantly produced at 30 °C, with FB_1_ exhibiting the highest concentrations under these conditions across all moisture contents, reaching a maximum of 1850 µg/kg. Fumonisin production was most pronounced under lower moisture conditions and was largely suppressed at temperatures ≥35 °C, irrespective of moisture level. At 40 °C, FB_1_ and FB_2_ were not detected at any moisture content.

**Table 3:** Effect of temperature and moisture content on fusarium mycotoxin production (µg/kg) in pig feed (Mean ± SD; n = 4).

Temperature (°C)	Moisture content (%)	DON	FUX	FB_1_	FB_2_	OTA	T2	HT2	ZEN	DAS
30	8	613.62 ± 58.47	522.97 ± 80.79	1850.04 ± 31.54	187.85 ± 56.17	9.442 ± 4.31	585.50 ± 14.75	383.28 ± 12.69	247.76 ± 42.62	413.19 ± 86.79
	10	ND	426.27 ± 10.99	932.78 ± 15.01	89.16 ± 1.25	ND	353.37 ± 39.87	ND	1.37 ± 0.09	ND
	12	ND	451.97 ± 46.71	1365.85 ± 1.07	116.22 ± 0.97	ND	ND	ND	1.86 ± 0.06	336.44 ± 4.18
	15	ND	467.14 ± 72.48	1154.64 ± 0.00	115.73 ± 0.04	ND	ND	ND	3.02 ± 10.07	
35	8	ND	321.32 ± 8.12	963.86 ± 0.45	ND	ND	ND	ND	4.35 ± 0.16	ND
	10	207.68 ± 0.26	425.25 ± 12.36	ND	115.71 ± 0.05	ND	ND	ND	2.38 ± 0.05	334.15 ± 3.51
	12	307.73 ± 0.23	439.40 ± 26.46	ND	115.68 ± 0.10	ND	ND	ND	1.37 ± 0.03	ND
	15	307.08 ± 0.00	319.29 ± 10.05	ND	115.75 ± 0.25	ND	ND	ND	0.45 ± 0.00	
37	8	418.43 ± 1.46	408.49 ± 32.97	ND	ND	3.97 ± 0.04	ND	ND	1.42 ± 0.04	ND
	10	308.46 ± 2.29	316.18 ± 6.17	ND	ND	5.90 ± 0.32	ND	ND	1.22 ± 0.09	ND
	12	408.23 ± 1.72	451.64 ± 31.76	ND	ND	ND	183.06 ± 2.11	ND	1.37 ± 0.03	332.78 ± 2.60
	15	509.16 ± 2.06	529.41 ± 10.85	ND	ND	5.96 ± 0.04	ND	ND	1.56 ± 0.09	375.70 ± 0.14
40	8	407.68 ± 0.65	432.85 ± 18.85	ND	ND	3.96 ± 0.02	ND	ND	2.40 ± 1.08	344.06 ± 0.52
	10	411.50 ± 1.39	468.97 ± 24.96	ND	ND	13.92 ± 0.01	ND	ND	1.04 ± 0.45	331.65 ± 0.34
	12	531.39 ± 3.51	473.05 ± 19.51	ND	ND	6.97 ± 0.06	183.07 ± 1.08	240.50 ± 17.34	1.22 ± 0.28	303.37 ± 0.53
	15	510.95 ± 1.55	454.55 ± 22.06	ND	ND	4.94 ± 0.03	ND	ND	1.45 ± 0.05	334.00 ± 1.72

ND, not detected.

**Table 4:** Effect of temperature and moisture content on fusarium mycotoxin production in broiler feed (µg/kg) (Mean ± SD; n = 4).

Temperature (°C)	Moisture Content (%)	DON	FUX	FB_1_	FB_2_	OTA	T2	HT2	ZEN
30	8	453.18 ± 29.23	4622.13 ± 86.34	278.95 ± 6.13	174.65 ± 0.13	24.15 ± 0.52	387.17 ± 88.43	274.16 ± 58.94	75.89 ± 0.06
	10	329.11 ± 0.87	2504.68 ± 18.81	182.05 ± 67.49	275.03 ± 1.18	23.48 ± 0.33	518.92 ± 7.10	1419.28 ± 28.63	68.84 ± 0.13
	12	431.16 ± 10.27	5255.13 ± 49.28	270.82 ± 37.21	312.25 ± 78.18	23.91 ± 0.47	405.52 ± 34.52	3061.33 ± 40.56	65.92 ± 2.00
	15	333.45 ± 10.23	3155.77 ± 13.13	222.79 ± 27.30	176.62 ± 1.29	24.04 ± 0.91	343.44 ± 23.00	1530.49 ± 30.68	66.94 ± 1.94
35	8	437.21 ± 0.00	3158.83 ± 90.82	368.52 ± 0.11	130.46 ± 5.88	ND	316.99 ± 18.24	1324.85 ± 61.34	60.81 ± 0.22
	10	440.95 ± 11.93	4577.20 ± 29.65	ND	140.53 ± 1.22	ND	398.20 ± 44.04	2619.02 ± 23.14	62.78 ± 0.56
	12	455.48 ± 0.00	3015.98 ± 15.79	ND	ND	ND	374.18 ± 12.36	848.85 ± 55.50	ND
	15	401.66 ± 0.00	1759.90 ± 66.04	ND	ND	ND	349.75 ± 18.49	1667.34 ± 49.23	62.94 ± 1.00
37	8	431.71 ± 0.25	4853.69 ± 27.19	ND	ND	ND	59.79 ± 0.00	ND	68.72 ± 0.25
	10	443.33 ± 3.00	2934.37 ± 15.79	ND	ND	ND	ND	ND	66.94 ± 0.33
	12	503.50 ± 55.86	2801.48 ± 29.02	ND	ND	ND	ND	ND	67.95 ± 0.98
	15	ND	2902.82 ± 69.27	ND	122.48 ± 0.54	ND	ND	ND	68.82 ± 0.18
40	8	ND	2867.21 ± 56.41	ND	130.51 ± 2.80	ND	60.40 ± 1.20	ND	77.94 ± 0.00
	10	443.75 ± 1.00	3013.72 ± 17.73	ND	ND	ND	ND	ND	72.02 ± 0.00
	12	426.00 ± 12.30	2551.90 ± 59.25	398.52 ± 2.66	ND	ND	ND	ND	68.79 ± 0.14
	15	ND	3493.76 ± 15.46	ND	ND	ND	ND	ND	69.80 ± 0.09

ND, not detected.

Deoxynivalenol production showed a strong temperature dependence, with the highest concentrations observed at 30 °C, particularly at lower moisture content. DON concentrations declined as temperature increased. At 35 °C, DON was consistently detected at 10-15% moisture content, with concentrations ranging from 207.01 to 307.74 µg/kg. At 37 °C, DON and FUX were consistently detected across all moisture levels, with DON concentrations ranging from 418.43 to 509.17 µg/kg. Similarly, at 40 °C, DON and FUX remained consistently present across all moisture levels. Detectable levels of ZEN were primarily observed at higher temperatures and intermediate moisture contents with consistent concentrations of average 1.36 µg/kg. In contrast, OTA and the trichothecenes (T-2 and HT-2 toxins) were detected occasionally and did not display a consistent interaction pattern between temperature and moisture content. OTA was detected at low concentrations at 37 °C (3.91 - 5.96 µg/kg) and 40 °C (3.96 - 13.92 µg/kg) across moisture contents. T-2 and HT-2 toxins were detected only at 12% moisture content, with high concentrations of 183.01 µg/kg and 240.51 µg/kg, respectively. Additionally, DAS was detected only at 12 - 15% moisture content, with concentrations ranging from 332.78 to 413.79 µg/kg.

**Table 4** shows the production in broiler feed, where DON production showed a strong interaction pattern, particularly at lower temperatures. At 30 °C, DON concentrations varied substantially with moisture content, with elevated levels observed at 8% and 12% moisture, while markedly reduced concentrations occurred at 10% and 15%. As the temperature increased to 35-37 °C, DON levels remained relatively high across most moisture levels but exhibited reduced sensitivity to moisture variation. At 40 °C, DON production became inconsistent and was undetectable at certain moisture contents, indicating that moisture effects on DON biosynthesis were temperature-dependent in broiler feed. Fusarenone-X was consistently detected across all temperature and moisture combinations, but its concentration was strongly influenced by their interaction. The highest FUX levels were recorded at 30 °C, particularly at 12% moisture, after which concentrations declined with increasing temperature. However, the magnitude of this decline varied by moisture level, demonstrating that FUX production in broiler feed was not governed by temperature alone but by the combined effect of both environmental factors. Fumonisins (FB_1_ and FB_2_) exhibited a pronounced interaction dominated by temperature sensitivity. At 30 °C, both FB_1_ and FB_2_ were detected at appreciable levels, with concentrations strongly influenced by moisture content. In contrast, fumonisin production was largely suppressed at temperatures ≥35 °C regardless of moisture level, indicating that moisture-driven effects on fumonisin biosynthesis occurred only under permissive temperature conditions in broiler feed.

**Figure 2:**
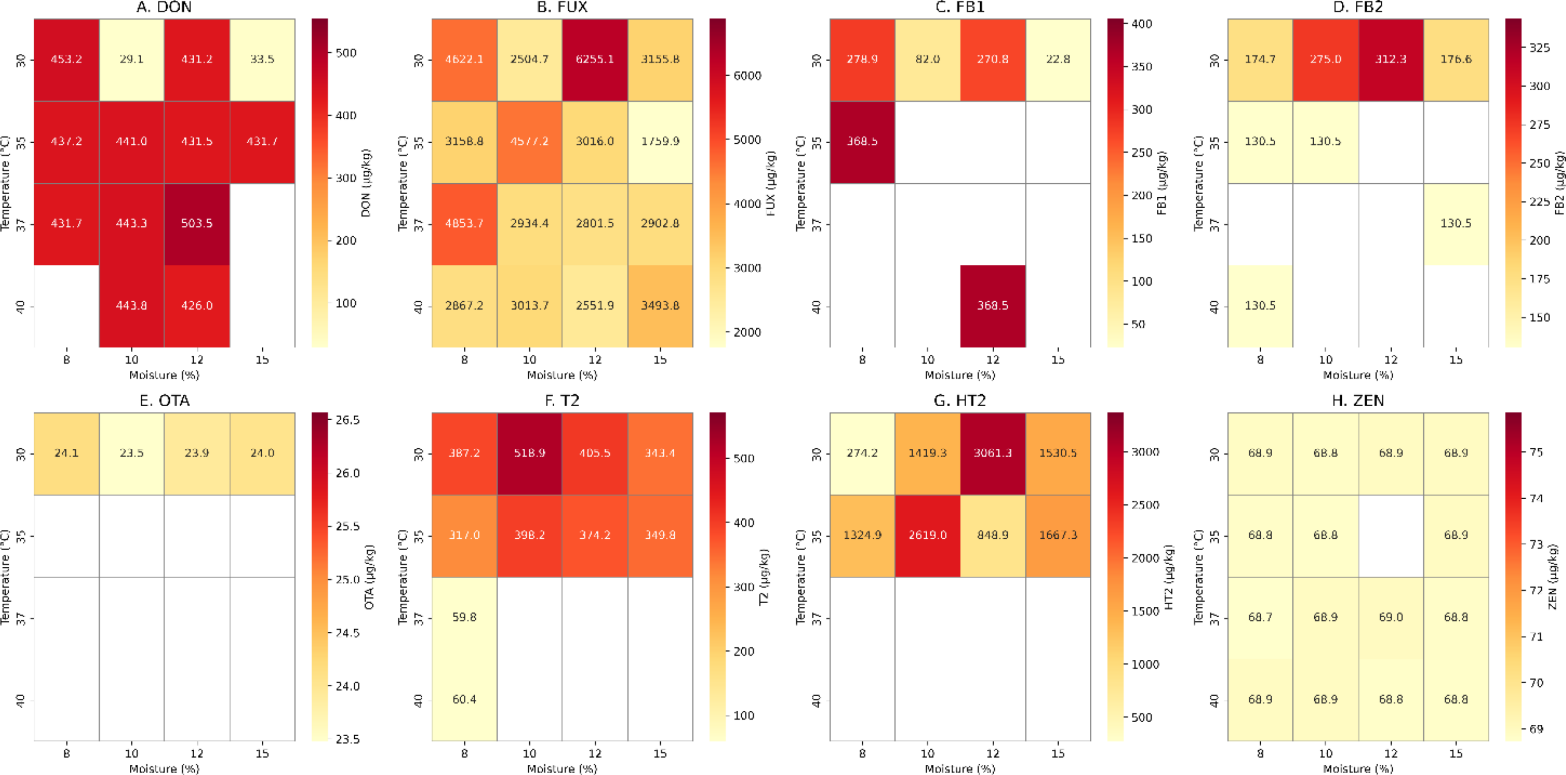
Heatmaps showing fusarium-related mycotoxin concentrations in pig and broiler feed across temperature and moisture combinations. (Heatmaps illustrate the levels of eight mycotoxins [**(A)** DON, **(B)** FUX, **(C)** FB_1_, **(D)** FB_2_, **(E)** OTA, **(F)** T-2 toxin, **(G)** HT-2 toxin, **(H)** ZEN] measured in feed incubated at four temperatures (30, 35, 37, 40 °C) and four moisture contents (8%, 10%, 12%, 15%) for 21 days. Color gradients (yellow → red) represent increasing concentrations (µg/kg), with annotated values shown in each cell. Not detected (ND) values are left blank. These heatmaps highlight the interactive effects of temperature and moisture on mycotoxin production and allow comparison of toxin-specific response patterns, revealing conditions that favor or suppress individual and co-occurring fusarium toxins).

Zearalenone showed minimal variation across temperature and moisture treatments, with relatively stable concentrations detected under most conditions, suggesting a weak interaction effect and greater environmental stability in broiler feed matrices. Trichothecenes T-2 and HT-2 toxins displayed moisture-dependent variability at 30 °C, particularly at intermediate moisture levels, but were sporadically detected or absent at higher temperatures, indicating sensitivity to combined temperature and moisture stress. Ochratoxin A was detected only at lower temperatures and was absent under most high-temperature conditions, limiting the expression of a consistent interaction pattern. **Figure 2** shows mycotoxin concentrations in pig and broiler feed across temperature and moisture combinations using heatmaps.

## 4 Discussion

The present study demonstrates that temperature and moisture play a critical role in fusarium-related mycotoxin production in both pig and broiler feed, with distinct toxin profiles emerging under different environmental conditions. Similar environmental dependencies have been widely reported for *Fusarium spp*, where secondary metabolite biosynthesis is strongly regulated by temperature, water activity, and substrate composition (Magan et al., 2011; Marin et al., 2013).

In pig feed, fumonisins (FB_1_ and FB_2_) were most prominently produced at 30 °C, particularly under lower moisture conditions, with FB_1_ reaching peak concentrations. These findings are consistent with previous studies showing that fumonisin production by *Fusarium verticillioides* is optimal at moderate temperatures (25 - 30 °C) and relatively low water activity (aw 0.95-0.98) (Medina et al., 2013). The marked suppression of fumonisin production at temperatures above 35 °C, regardless of moisture content, suggests that temperature exerts a dominant regulatory effect on fumonisin biosynthetic gene expression, as reported in earlier molecular and ecological study of (Fanelli et al., 2013). However, in broiler feed, fumonisin production was also largely restricted to lower temperatures, although concentrations were generally lower and more variable than those observed in pig feed. This difference may be attributed to variations in feed composition, including starch availability, lipid content, and micronutrient composition, all of which influence fungal growth and secondary metabolism (Wokorach et al., 2021). Pig feed generally contains a higher starch content due to its cereal-based formulation, which may provide a favorable substrate for fungal growth and subsequent mycotoxin production, whereas broiler feed is characterized by a higher crude protein content that may influence fungal metabolism and toxin biosynthesis differently.

Deoxynivalenol production showed a strong temperature-dependent pattern across both feed matrices. In pig feed, DON concentrations were highest at 30 °C, particularly under lower moisture conditions, and declined as temperature increased. This agrees with previous reports indicating that DON biosynthesis is favored at moderate temperatures and is sensitive to thermal stress (Kolawole et al., 2021; Huang et al., 2023). In contrast, in broiler feed, DON remained consistently detectable across a wider temperature range (35-40 °C), especially at intermediate moisture contents. This sustained production under elevated temperatures suggests that substrate composition may buffer environmental stress and support trichothecene biosynthesis, a phenomenon previously described in cereal-based matrices (Zhao et al., 2025).

Fusarenone-X was consistently detected across all temperature and moisture combinations in both pig and broiler feed and occurred at substantially higher concentrations than other fusarium-related mycotoxins. This ubiquitous presence is consistent with earlier studies reporting that fusaric acid production is less sensitive to environmental fluctuations than other mycotoxins and often co-occurs with DON and fumonisins (Bacon et al., 1996). Importantly, fusaric acid has been shown to enhance the toxicity of other *fusarium spp* mycotoxins, even at relatively low concentrations, raising concerns about synergistic effects in contaminated feeds (Ogunbo et al., 2007; López-díaz et al., 2018). Stable concentrations of ZEN were observed across temperature and moisture content in broiler feed, whereas greater variability was observed in pig feed. The persistence of ZEN at higher temperatures contrasts with the temperature sensitivity observed for fumonisins and reflects differences in the regulation of polyketide-derived estrogenic compounds (Ryu et al., 2002; Zhang et al., 2015). Despite its relatively low concentrations, the consistent presence of ZEN is relevant from a feed safety perspective due to its cumulative estrogenic effects, particularly in pigs and poultry (EFSA et al., 2017).

Ochratoxin A and the type A trichothecenes (T-2 and HT-2 toxins) in both feed matrices did not exhibit a consistent temperature-moisture interaction pattern. Their intermittent occurrence is consistent with previous reports indicating that these toxins are produced by a narrower range of fungal species and are more sensitive to environmental stressors than DON or fumonisins (Eskola et al., 2020). In broiler feed, the detection of T-2 and HT-2 toxins primarily at 12% moisture content suggests that moderate moisture levels may be critical for their biosynthesis, as reported in controlled storage studies (Magan et al., 2011). Although *F. graminearum* was the fungal species intentionally used for inoculation in this study, it should be noted that some of the detected mycotoxins may not be exclusively associated with this species. Certain toxins could originate from background contamination.

Overall, the comparative analysis of pig and broiler feed indicates that temperature is the primary driver of fusarium-related mycotoxin production, while moisture content acts as a modifying factor whose impact depends on feed matrix composition. Lower temperatures favored the co-occurrence of multiple mycotoxins, whereas higher temperatures selectively supported DON and fusaric acid production, fumonisin being highly temperature-restricted, and ZEN displaying relative stability across environmental conditions. These findings are consistent with current ecological models of mycotoxin biosynthesis and emphasize the importance of feed-specific storage and management strategies (Marin et al., 2013; Eskola et al., 2020). It further emphasizes the importance of controlling temperature and moisture content during feed storage to reduce mycotoxin risk. The study further confirms that, in addition to the two selected abiotic factors evaluated, other environmental variables such as pH and light exposure were not considered, yet these factors may significantly influence mycotoxin production by fungi (Magan and Aldred, 2007; Bennett and Klich, 2003). Environmental pH can affect enzymatic activity and fungal metabolism, while light conditions may regulate secondary metabolite gene expression (Paterson and Lima, 2010). Furthermore, it is important to recognize the influence of feed composition on mycotoxin production. The nutritional matrix, including carbohydrate source, protein content, lipid composition, and micronutrient availability, can modulate fungal growth dynamics and the activation of toxin biosynthesis pathways (Payne and Brown, 1998). The findings therefore reinforce that mycotoxin production is a multifactorial process dependent not only on specific abiotic conditions but also on the substrate (matrix) composition and biotic interactions within the environment. This highlights the complexity of predicting mycotoxin contamination and the necessity of adopting an integrated approach when assessing fungal toxin risk in feed systems (International Agency for Research on Cancer (IARC), 2012; Magan and Aldred, 2007).

## 5 Conclusion, limitations, and future perspectives

This study demonstrated that temperature and moisture content are key determinants of mycotoxin production in pig and broiler feed, with distinct toxin profiles and concentrations observed across different environmental conditions. Temperature emerged as the primary driver of mycotoxin occurrence, while moisture content acted as a modifying factor influencing toxin diversity and magnitude in a feed-dependent manner. Lower temperatures, particularly around 30 °C, promoted the co-production of multiple mycotoxins, including Fumonisin, deoxynivalenol, fusarenone-X, zearalenone, and trichothecenes, whereas higher temperatures generally suppressed fumonisin production while allowing sustained production of deoxynivalenol and fusaric acid. Comparative evaluation of pig and broiler feed highlighted the influence of feed matrix composition on fungal metabolism and toxin biosynthesis. The frequent co-occurrence of multiple mycotoxins across temperature and moisture combinations underscores the risk of chronic multi-toxin exposure in livestock, which may result in additive or synergistic adverse effects on animal health and performance. Despite these findings, several limitations are acknowledged. The study was conducted under controlled experimental conditions, which may not fully replicate the complexity and variability of commercial feed storage environments. Only a defined range of temperatures and moisture contents was evaluated, and other influential factors such as storage duration, oxygen availability, fungal community dynamics, and feed formulation variability were not assessed. In addition, mycotoxin production was evaluated based on concentration measurements, without direct assessment of fungal biomass or gene expression, which could provide further insight into regulatory mechanisms underlying toxin biosynthesis.

## Data Availability

The original contributions presented in the study are included in the article/supplementary material. Further inquiries can be directed to the corresponding author.
